# Lipoxin A4 and its analog suppress inflammation by modulating HMGB1 translocation and expression in psoriasis

**DOI:** 10.1038/s41598-017-07485-1

**Published:** 2017-08-02

**Authors:** Xinxin Liu, Xin Wang, Xiaoru Duan, Devesh Poorun, Juntao Xu, Song Zhang, Lu Gan, Mengwen He, Ke Zhu, Zhangyin Ming, Feng Hu, Hongxiang Chen

**Affiliations:** 1 Department of Dermatology, Union Hospital, Tongji Medical College, Huazhong University of Science and Technology, Wuhan, 430022 China; 2 Cutaneous Biology Research Center, Department of Dermatology, Massachusetts General Hospital, Harvard Medical School, Building 149, 13th Street Charlestown, Boston, MA 02129 USA; 3 Department of Pharmacology, Tongji Medical College, Huazhong University of Science and Technology, Wuhan, 430030 China

## Abstract

Psoriasis is a chronic inflammatory skin disease that affects 2–3% of the global population, and there is still no known possibility of a cure. Lipoxin A4 (LXA4), an endogenous lipoxygenase-derived eicosanoid mediator, has potent dual pro-resolving and anti-inflammatory properties. BML-111 (5(S)-6(R)-7-trihydroxyheptanoic acid methyl ester), a lipoxin receptor agonist, has been previously confirmed to be equivalent to LXA4 in the anti-inflammatory processes. High mobility group box 1 (HMGB1) serves as an inflammatory cytokine when secreted extracellularly in psoriatic lesions and is involved in the development of psoriasis. Therefore, we investigated the effects of LXA4 and BML-111 on the HMGB1 signaling cascade and inflammation in lipopolysaccharide (LPS)-induced keratinocytes and imiquimod (IMQ)-induced psoriasiform dermatitis in mice. In the present study, we found that treatment with BML-111 attenuated the development of IMQ-induced psoriasiform dermatitis. Furthermore, treatment with BML-111 and LXA4 inhibited HMGB1 translocation from the nucleus to cytoplasm and downregulated the expression of toll-like receptor 4 (TLR4), receptor for advanced glycation end products (RAGE), p-ERK1/2, nuclear NF-κB p65, and proinflammatory cytokines *in vivo* and *in vitro*. Our findings indicate that LXA4 and its analog may be potential therapeutic candidates for psoriasis because of their ability to modulate the translocation and expression of HMGB1.

## Introduction

Psoriasis is a chronic inflammatory skin disease that affects 2–3% of the global population^1^. It is characterized by the hyperproliferation of keratinocytes, dilation and growth of dermal capillary vasculature, and infiltration of T lymphocytes and neutrophils into the dermis and epidermis. Within the skin as well as in the circulation of psoriasis patients, the production of numerous cytokines and chemokines is increased^2, 3^. However, the exact pathogenesis of psoriasis is not yet fully understood, and a complete cure is lacking.

High mobility group box 1 (HMGB1) was so named because of its characteristic rapid mobility in polyacrylamide gel electrophoresis and was first described as a DNA-binding protein. HMGB1 is a nuclear protein that is expressed in almost all cells, and it binds to DNA where it acts as a transcriptional regulatory factor^4, 5^. It is now known that HMGB1 is also a proinflammatory cytokine that can be translocated to the cytosol and then secreted into the extracellular space from various cells in response to injury, infection or other inflammatory stimuli^6^. There are two main pathways, passive and active, by which HMGB1 translocation occurs. In the passive process, HMGB1 is released by necrotic cells. On the other hand, the active process occurs when cells are stimulated by inflammatory mediators, such as lipopolysaccharides (LPS)^7, 8^. Once released, HMGB1 binds to receptors, including receptor for advanced glycation end products (RAGE) and toll-like receptors (TLRs), such as TLR2 and TLR4. This leads to signal transduction that elicits cellular responses including upregulation of proinflammatory cytokines (e.g., TNF-α, IL-1β, and IL-8) and acute inflammation^9, 10^. Recent extensive work has shed light on the role of HMGB1 in the pathogenesis of many types of inflammatory and autoimmune diseases^10, 11^. Elevated serum levels of HMGB1 have been found in patients with psoriasis, which are significantly decreased after treatment. The cytoplasmic expression of HMGB1 has been found in the skin lesions of psoriasis patients^12, 13^. A recent study has demonstrated that HMGB1 secreted from keratinocytes can facilitate the expression and secretion of IL-18 by an autocrine mechanism, thus contributing to the development of psoriasis^14^. These studies suggest that HMGB1 may play a critical role in the pathogenesis of psoriasis.

Lipoxin A4 (LXA4), an endogenous lipoxygenase-derived eicosanoid mediator, is produced from arachidonic acid. LXA4 exerts potent dual anti-inflammatory and pro-resolving effects on various cell types. LXA4 has been found to suppress leukocyte-mediated injury, promote chemotaxis of monocytes and phagocytosis of apoptotic neutrophils^15, 16^, and inhibit the production of proinflammatory cytokines and cell proliferation. Our previous studies have suggested that LXA4 inhibits the growth of normal human epidermal keratinocytes (NHEKs) and their inflammatory cytokine/chemokine production, while its inhibitory effects might be associated with the cyclinD1/P16INK4A, ERK1/2, and NF-κB signal transduction pathways^17, 18^. BML-111 (5(S)-6(R)-7-trihydroxyheptanoic acid methyl ester) is a commercially available synthetic lipoxin (LX) receptor agonist, which is even more potent and stable than the original LX molecule. BML-111 has extensive biological actions; it has demonstrated potent pro-resolving and anti-inflammatory effects in mouse models of arthritis^19^. BML-111 has been previously confirmed to be equivalent to LXA4 in the inhibition of inflammatory processes^20, 21^.

Previous studies have shown that application of imiquimod (IMQ) to the skin of mice induces lesions closely resembling psoriasis in human, which are both phenotypically and histologically dependent on the IL-23/IL-17 axis^22^. Therefore, IMQ-induced skin inflammation in mice represents a useful model of human psoriasis^23^. Given the importance of HMGB1 in the initiation and progression of inflammatory responses, the aim of this study was to explore whether LXA4 and its analog could inhibit inflammatory cytokines, especially the expression and secretion of HMGB1 and to examine the molecular mechanisms underlying this effect. Thus, in *in vitro* experiments involving NHEKs, LXA4 was applied, while in *in vivo* experiments involving IMQ-induced psoriasis-like skin in mouse model, BML-111 was used to mimic the effects of LXA4.

## Results

### BML-111 alleviated the morphological and histological changes of IMQ-induced psoriasiform dermatitis in mice

**Figure 1:**
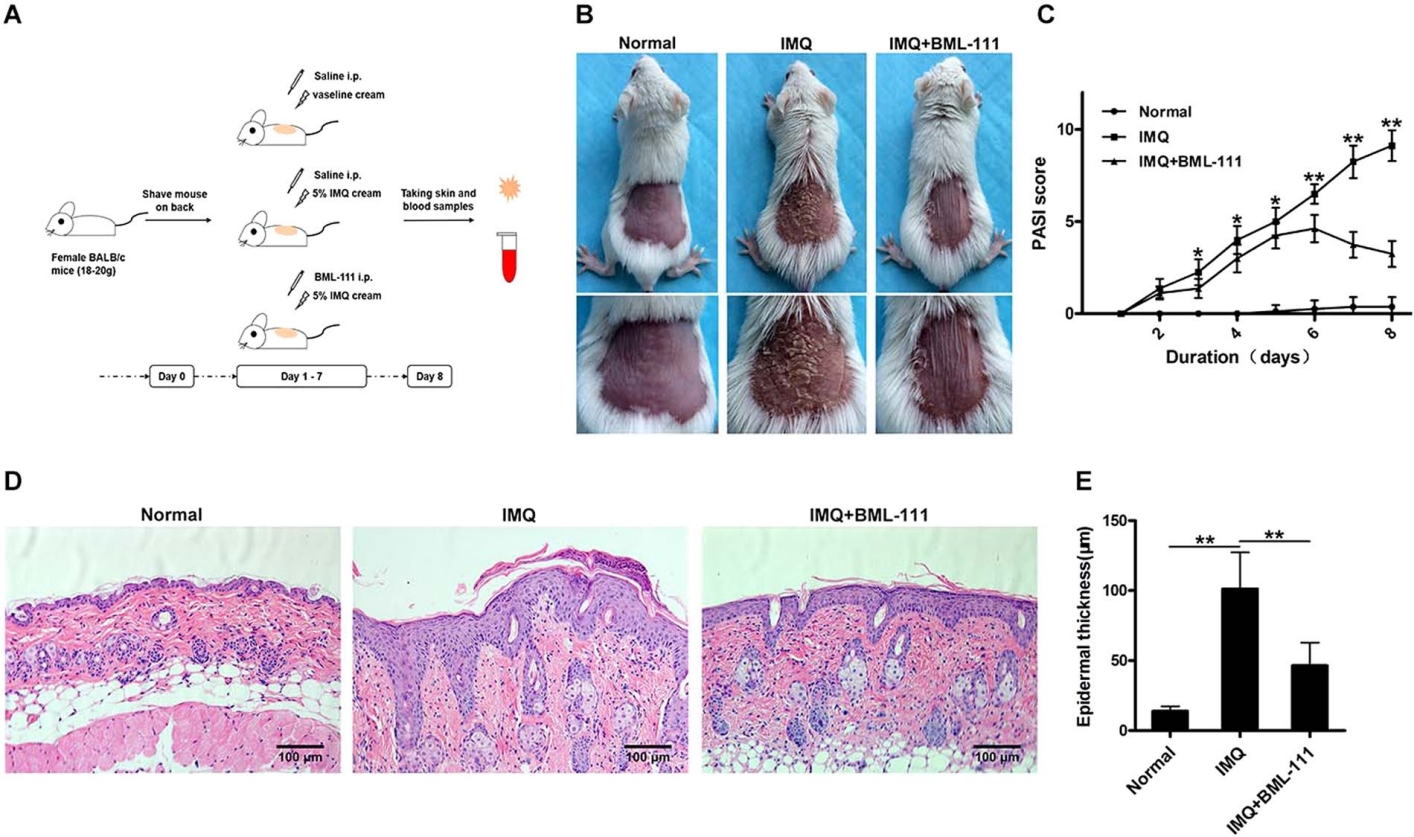
BML-111 improves the morphological and histological features of IMQ-induced psoriasiform dermatitis in mice. (**A**) Schematic representation of the animal experiment protocol. (**B**) Representative macroscopic views of the dorsal skin of BALB/c mice following continuous treatment for 8 days. (**C**) Epidermal erythema, scaling and thickening of the dorsal skin were evaluated daily; the clinical PASI score was calculated by adding the scores of the three separate criteria (range from 0 to 12). (**D**) HE staining of cross-sectional slices from the dorsal skin of the three groups of mice. (**E**) Epidermal thickness of the dorsal skin on day 8. **p < 0.01 and *p < *0*.*05*, compared with the mice in the IMQ group. Each bar represents the mean ± SD (n = 8).

Compared with the normal group, the dorsal skin of mice treated with IMQ exhibited signs of erythema, scaling, and thickening after 2 days, and the lesions gradually increased with extended IMQ administration. During the same period, mice pretreated with BML-111 exhibited decreased erythema, thin scales, smooth skin, and reduced thickening, and the overall skin lesions were significantly reduced (Fig. 1B). The average Psoriasis Area and Severity Index (PASI) score of each group is plotted in Fig. 1C. Mice in the normal group treated daily with Vaseline exhibited no significant changes in the PASI scores. Interestingly, mice in the IMQ + BML-111 group, which were pretreated with BML-111, exhibited lower PASI scores than those in the IMQ group.

The histological results from the IMQ group showed increased epidermal hyperplasia, acanthosis, and parakeratosis. Munro micro abscesses were formed, and infiltration of inflammatory cells into the dermal layer was observed. BML-111 notably improved histological injuries in the IMQ + BML-111 group, and hematoxylin-eosin (HE) staining exhibited smoother epidermis, lower degree of parakeratosis, and lower extent of epidermal thickening than those in the IMQ group (Fig. 1D). The thickness of the epidermis in the IMQ group was significantly increased (101.20 ± 9.25 μm), compared with that of the normal group (13.99 ± 1.21 μm). BML-111 decreased epidermal thickness in the IMQ-treated mice (46.58 ± 5.77 μm) (Fig. 1E).

### BML-111 attenuated the translocation and expression of HMGB1 in IMQ-induced psoriasiform dermatitis in mice

**Figure 2:**
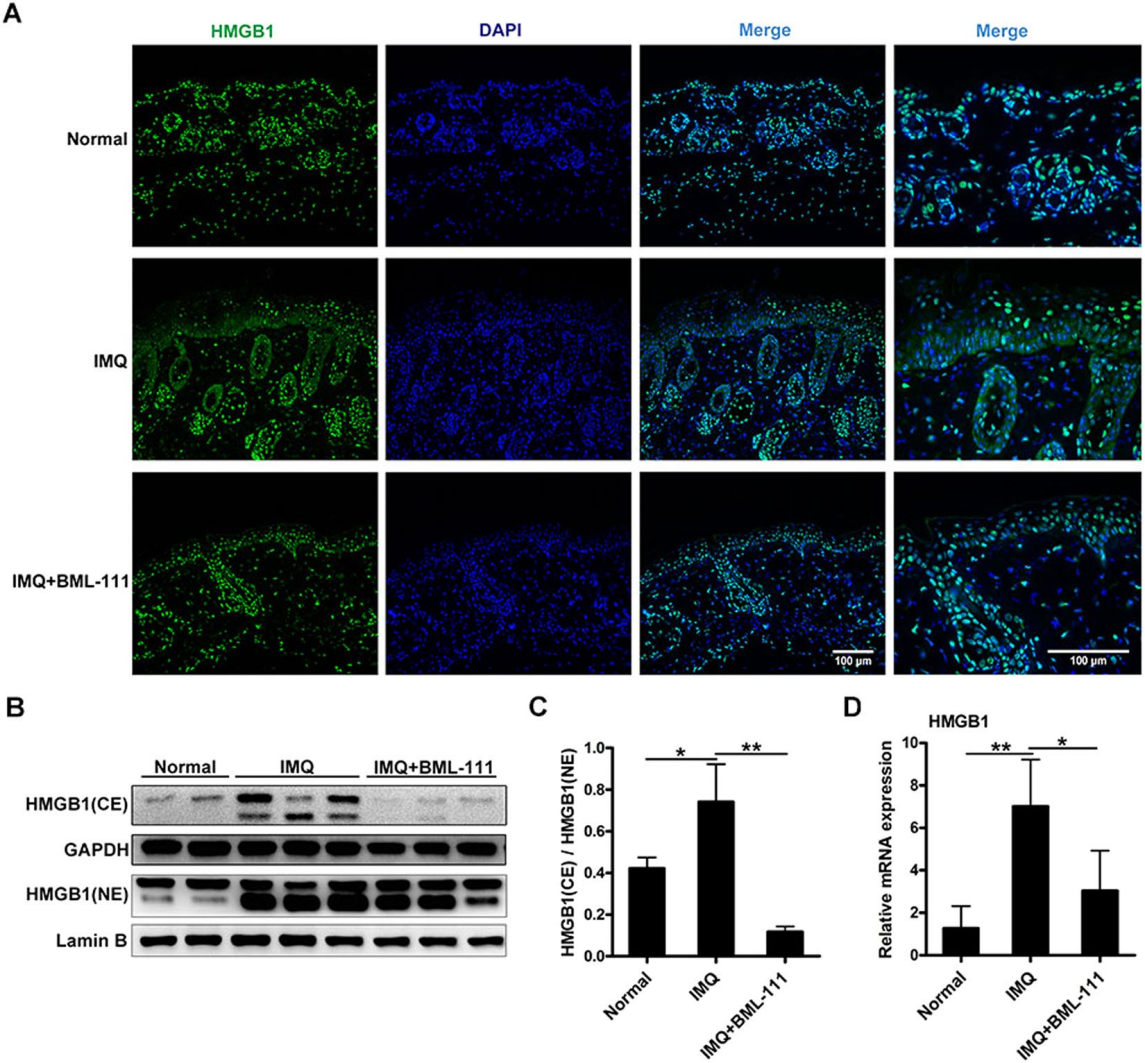
BML-111 attenuates the translocation and expression of HMGB1 in IMQ-induced psoriasiform dermatitis in mice. (**A**) Immunofluorescence staining of HMGB1 (green) in skin lesions of mice. Nuclei were counterstained with DAPI (blue). (**B**) Cytoplasmic extract (CE) and nuclear extract (NE) from the epidermal cells in the lesional skin of mice were used for western blots analysis of the indicated proteins. Full-length blots are presented in Suppl. Figure S1. (**C**) Ratio of HMGB1 expression in CE to NE was assessed by the quantification of relative optical density. (**D**) HMGB1 mRNA levels in separated epidermis of mouse skin biopsies were evaluated using PCR. The results are shown as the mean ± SD. **p < 0.01 and *p < *0*.*05*, compared to mice treated with IMQ. All experiments were conducted thrice, and the representative results are shown.

We further explored the possible mechanisms underlying the anti-psoriatic effects of BML-111. Studies haves indicated that HMGB1 acts as a proinflammatory cytokine and is involved in the pathogenesis of psoriasis. Thus, we investigated whether BML-111 treatment could inhibit HMGB1 expression in our study. The distribution of HMGB1 in the lesions of mice was detected by immunofluorescence. As shown in Fig. 2A, HMGB1 was expressed both in the epidermis and dermis. The expression of HMGB1 in the skin specimens of the normal group was almost completely restricted to the nucleus in the epidermis. However, abundant cytoplasmic expression of HMGB1 was observed in the epidermis of lesional skin of the IMQ group, but this overexpression was decreased by pretreatment with BML-111 in the IMQ + BML-111 group (Fig. 2A).

To better establish the altered distribution of HMGB1 in the lesional skin of mice, western blotting was performed to investigate the expression of HMGB1 in the cytoplasmic and nuclear proteins. The data obtained from western blotting (Fig. 2B and Suppl. Figure S1) were in line with the findings by immunofluorescence. HMGB1 was seen in the nuclear extract (NE) and was absent in the cytoplasmic extract (CE) in the normal group. Moreover, cytoplasmic levels of HMGB1 were significantly increased by IMQ treatment but were markedly suppressed by BML-111 treatment. The ratio of cytoplasmic to nuclear HMGB1 expression was measured. As shown in Fig. 2C, the ratio in the IMQ + BML-111 group was significantly lower than that in the IMQ group. In addition, we also determined the mRNA expression of HMGB1 in skin lesions, and the results showed that IMQ induced a marked increase in HMGB1 mRNA expression, which was markedly decreased by BML-111 pretreatment (Fig. 2D).

### Effects of BML-111 on the HMGB1/RAGE and HMGB1/TLR4 signaling in IMQ-induced psoriasiform dermatitis in mice

**Figure 3:**
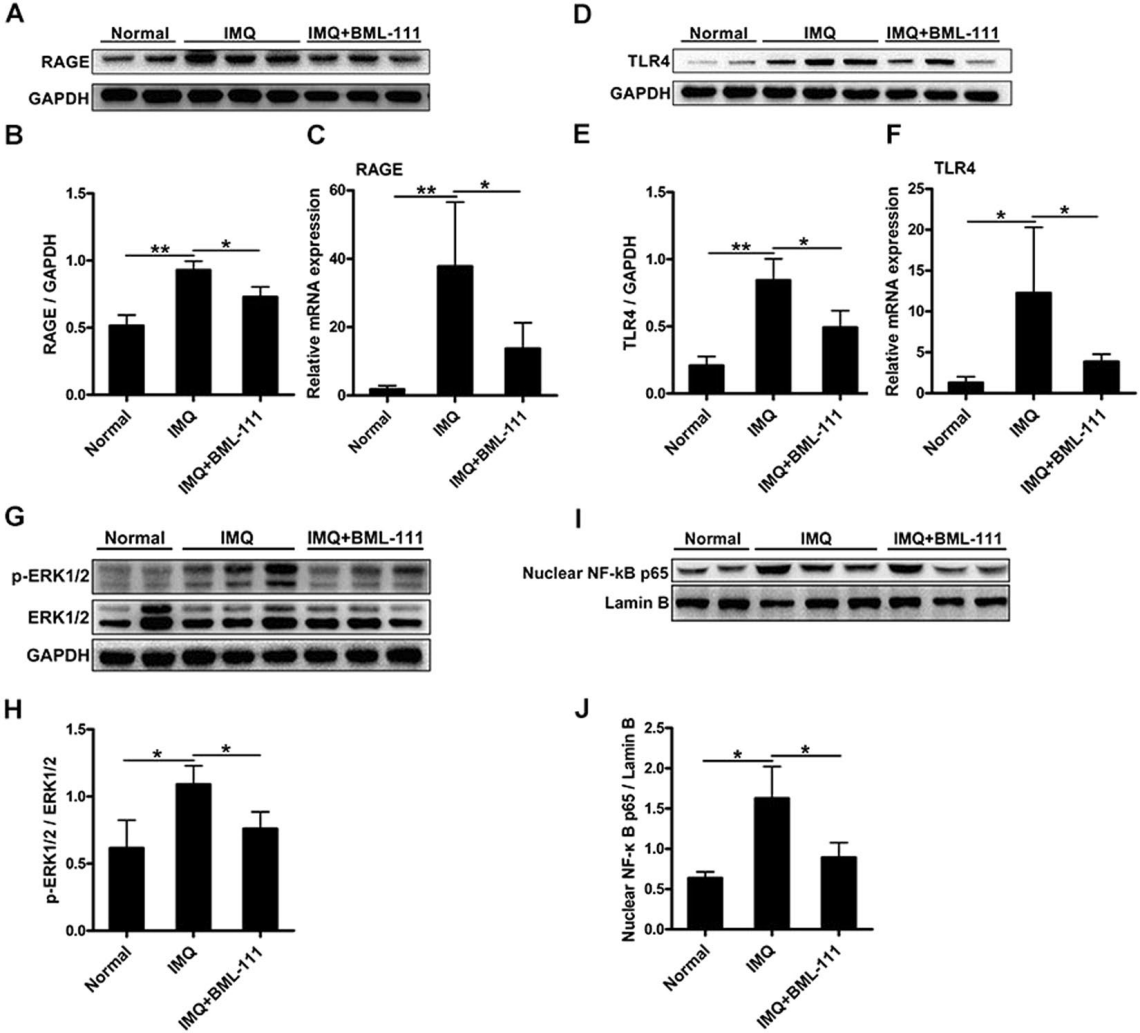
BML-111 suppresses the expression of proteins related to RAGE and TLR4 signaling in IMQ-induced psoriasiform dermatitis in mice. Protein levels of RAGE (**A**,**B**), TLR4 (**D**,**E**), ERK1/2 and p-ERK1/2 (**G**,**H**), and nuclear NF-κB p65 (**I**,**J**) were analyzed by western blotting. Full-length blots are presented in Suppl. Figures S2–S5. mRNA levels of RAGE (**C**) and TLR4 (**F**) were analyzed in the three groups of mice. The results are shown as the mean ± SD. **p < 0.01 and *p < *0*.*05*, compared to mice treated with IMQ. All experiments were conducted thrice, and the representative results are shown.

Previous studies have reported that the binding of HMGB1 to RAGE and TLR4 contributes to immune and inflammatory responses through downstream NF-κB and MAPK signaling^24, 25^. Therefore, we performed western blot analysis to measure the expression levels of RAGE, TLR4, p-ERK1/2, and nuclear NF-κB p65 in the skin homogenates of mice. Our results demonstrated that the protein levels of RAGE (Fig. 3A,B and Suppl. Figure S2), TLR4 (Fig. 3D,E and Suppl. Figure S3), p-ERK1/2 (Fig. 3G,H and Suppl. Figure S4), and nuclear NF-κB p65 (Fig. 3I,J and Suppl. Figure S5) were significantly increased in the IMQ group compared with those in the normal group. These changes were significantly attenuated by BML-111 treatment. The mRNA expression of RAGE and TLR4 in the skin lesions was examined by PCR. As shown in Fig. 3C and F, the increased mRNA expression of RAGE and TLR4 induced by IMQ was significantly suppressed by BML-111 pretreatment.

### BML-111 improved IMQ-induced changes in proinflammatory cytokines

**Figure 4:**
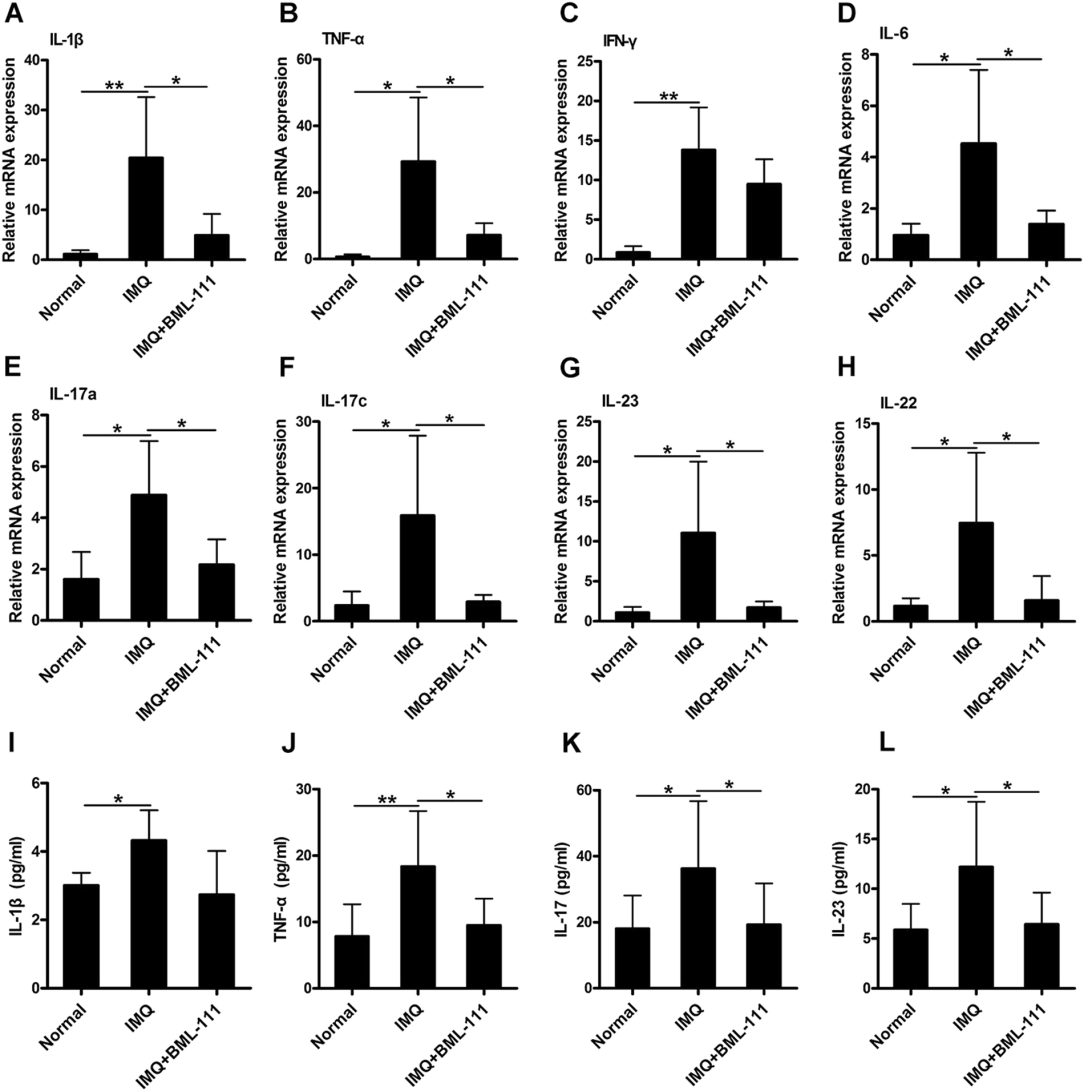
BML-111 decreases the inflammatory cytokines expression in IMQ-induced psoriasiform dermatitis in mice. Real-time quantitative PCR was performed to analyze the mRNA expression of IL-1β (**A**), TNF-α (**B**), IFN-γ (**C**), IL-6 (**D**), IL-17a (**E**), IL-17c (**F**), IL-23 (**G**), and IL-22 (**H**) in the skin biopsies from mice. The serum concentration of IL-1β (**I**), TNF-α (**J**), IL-17 (**K**) and IL-23 (**L**) in the collected serum on day 8 was determined by ELISA. The results are shown in mean ± SD. **p < 0.01 and *p < *0*.*05* when compared to mice treated with IMQ. All experiments were conducted thrice, and the representative results are shown.

The expression of proinflammatory cytokines in the skin lesions and serum samples of mice was investigated to evaluate the effects of BML-111 on IMQ-induced local and systemic inflammation. As shown in Fig. 4A–H, the mRNA expression of IL-1β, TNF-α, IFN-γ, IL-6, IL-17a, IL-17c, IL-23 and IL-22 was significantly increased in the IMQ group compared with that in the normal group, whereas treatment with BML-111 significantly inhibited the IMQ-induced increase in mRNA expression of these cytokines, except for IFN-γ. In addition, the increase in the serum levels of TNF-α, IL-17 and IL-23 in the IMQ group were notably decreased by BML-111 administration (Fig. 4J–L). Lower serum levels of IL-1β in the IMQ + BML-111 group than those in the IMQ group were observed, but the results were not statistically significant (*p* > *0*.*05*) (Fig. 4I).

### LXA4 suppressed the translocation and expression of HMGB1 in LPS-induced keratinocytes

**Figure 5:**
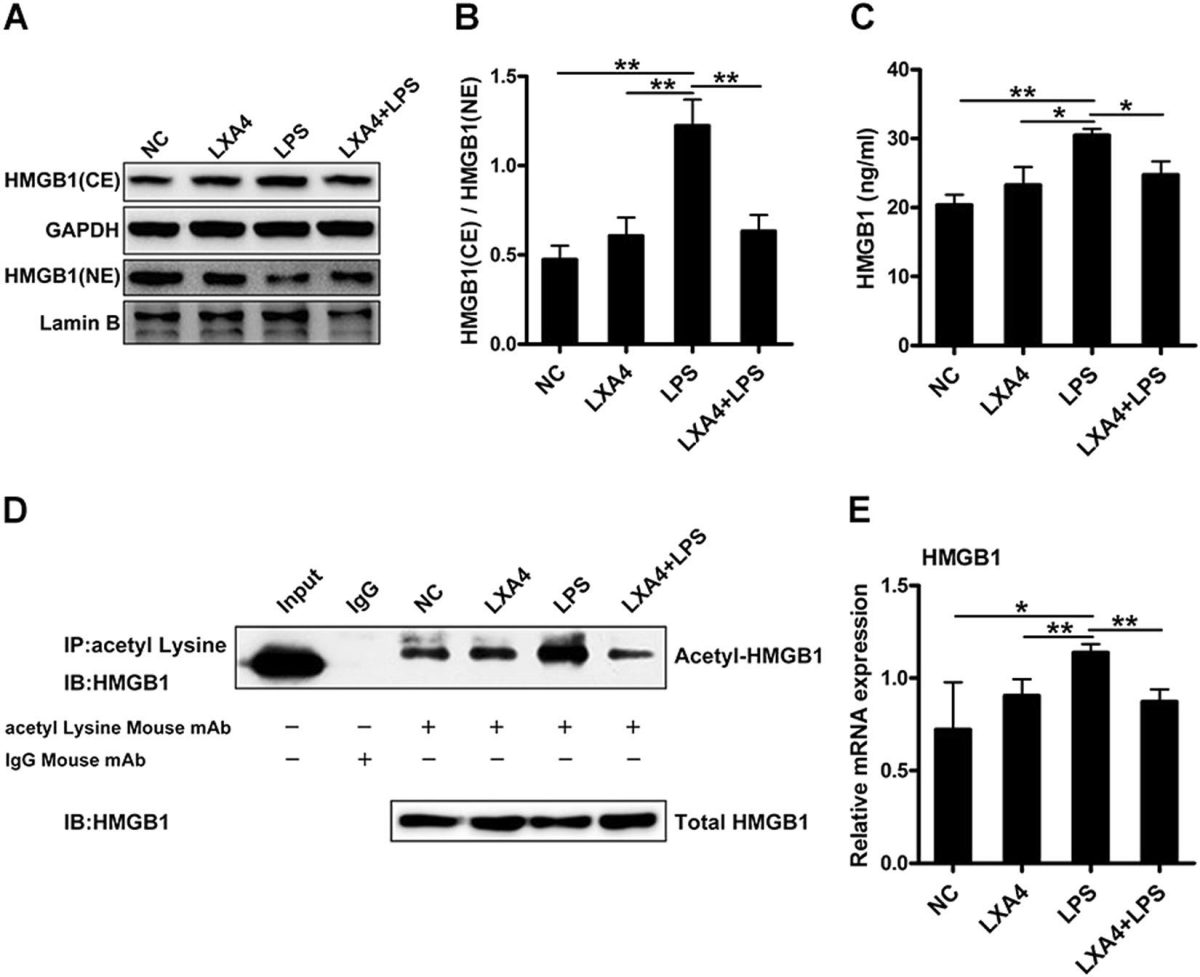
LXA4 pretreatment downregulates the LPS-induced secretion and expression of HMGB1 in keratinocytes. NHEKs were stimulated with LPS (10 μg/ml) with and without preincubation with LXA4 (100 nmol/l) for 30 minutes. (**A**) Western blot analysis shows specific bands for the expression of nuclear and cytoplasmic HMGB1 in NHEKs. Full-length blots are presented in Suppl. Figure S6. (**B**) Ratio of HMGB1 expression in CE to NE was assessed by the quantification of relative optical density. (**C**) The culture supernatant of NHEKs was obtained 24 hours after LPS challenge to determine HMGB1 concentration by ELISA. (**D**) Whole cell lysates of NHEKs in the four groups were immunoprecipitated with an anti-acetyl-lysine antibody and immunoblotted for HMGB1. Anti-mouse IgG was used as a negative control. Full-length blots are presented in Suppl. Figure S7. (**E**) NHEKs were collected after LPS stimulation to determine HMGB1 mRNA levels by PCR. The results are shown as the mean ± SD. **p < 0.01 and *p < *0*.*05*, compared to NHEKs treated with LPS without LXA4. All experiments were conducted thrice, and the representative results are shown.

In the next step, we explored whether LXA4 could inhibit HMGB1 secretion *in vitro*. We examined HMGB1 content by western blotting and the mRNA expression of HMGB1 by PCR in NHEKs following stimulation with LPS. As shown in Fig. 5A and Suppl. Figure S6, HMGB1 protein levels in the cytoplasm of NHEKs were induced by LPS, which were decreased after preincubation with LXA4 but decreased in the nucleus after stimulation with LPS. Furthermore, we measured the cytoplasmic to nuclear ratio of HMGB1 expression (Fig. 5B). NHEKs stimulated with LPS had a higher ratio compared with that in the other groups, which showed that LPS treatment significantly induced HMGB1 translocation from the nucleus to cytoplasm, but preincubation with LXA4 inhibited this effect. Cell culture supernatant was obtained after 24 hours of incubation to determine the concentration of HMGB1 by enzyme-linked immunosorbent assay (ELISA). As shown in Fig. 5C, HMGB1 secretion was increased by LPS, and it was clearly reduced by LXA4 pretreatment. Acetylation is a post-translation modification, and it appears to be critical for active HMGB1 secretion in many cells, such as macrophages and hepatocytes^26, 27^. To investigate the mechanism underlying the inhibitory effect of LXA4 on HMGB1 secretion from NHEKs, we evaluated the acetylation level of HMGB1 *in vitro*. Using co-immunoprecipitation from whole cell lysates of keratinocytes, we found that LPS could induce the acetylation of HMGB1, but the acetylated HMGB1 was significantly decreased by LXA4 preincubation (Fig. 5D and Suppl. Figure S7). The HMGB1 mRNA expression was significantly upregulated after LPS stimulation, which was markedly decreased by LXA4 preincubation (Fig. 5E). Altogether, these data indicate that LXA4 can inhibit the secretion and expression of HMGB1 *in vitro*.

### Effects of LXA4 on the HMGB1/RAGE and HMGB1/TLR4 signaling in LPS-induced keratinocytes

**Figure 6:**
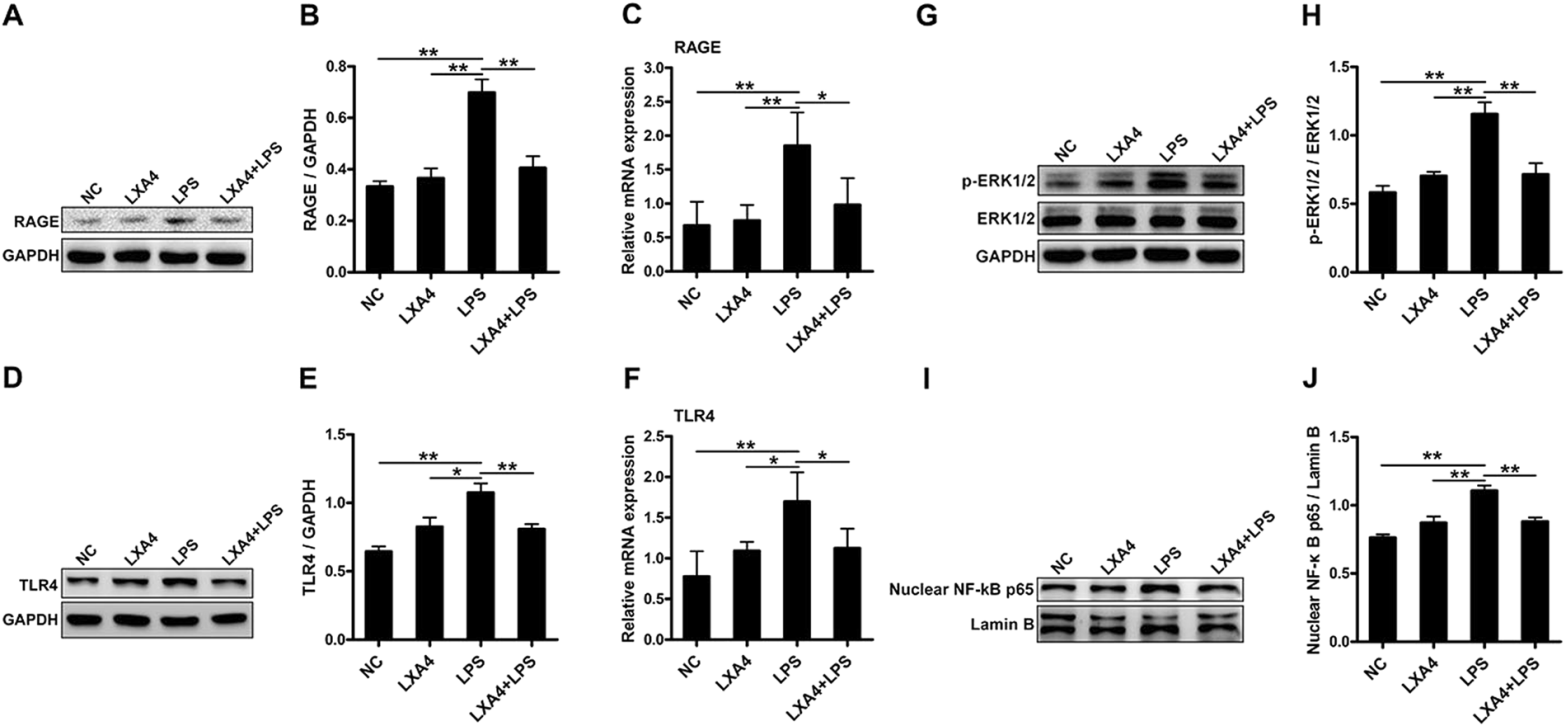
LXA4 inhibits the expression of the proteins related to RAGE and TLR4 signaling in keratinocytes. NHEKs were treated with LPS (10 μg/ml) for 24 hours with and without preincubation with LXA4 (100 nmol/l) for 30 minutes. Protein levels of RAGE (**A**,**B**), TLR4 (**D**,**E**), ERK1/2 and p-ERK1/2 (**G**,**H**), and nuclear NF-κB p65 (**I**,**J**) were analyzed by western blotting. Full-length blots are presented in Suppl. Figures S8–S11. mRNA levels of RAGE (**C**) and TLR4 (**F**) were analyzed by PCR. The results are shown as the mean ± SD. **p < 0.01 and *p < *0*.*05*, compared to NHEKs treated with LPS without LXA4. All experiments were conducted thrice, and the representative results are shown.

We further elucidated the effects of LXA4 on the downstream signaling factors induced by HMGB1 in NHEKs. After 24 hours of incubation in each treatment group, western blot analysis of RAGE, TLR4, p-ERK1/2, total ERK1/2, and nuclear NF-κB p65 proteins was performed. The results are shown in Fig. 6. A significant increase in the expression of RAGE (Fig. 6A,B and Suppl. Figure S8), TLR4 (Fig. 6D,E and Suppl. Figure S9), p-ERK1/2 (Fig. 6G,H and Suppl. Figure S10), and nuclear NF-κB p65 (Fig. 6I,J and Suppl. Figure S11) was noted in the cell samples that were stimulated with LPS. These effects were substantially inhibited by LXA4. In line with the protein levels, the mRNA expression of TLR4 and RAGE in NHEKs was clearly induced by LPS and suppressed by LXA4 (Fig. 6C,F).

## Discussion

Studies have shown that LXA4 can inhibit inflammatory responses in many diseases^19, 28, 29^. It is known that the etiologies of psoriasis are complex, and the additional mechanisms by which LXA4 confers protection in psoriasis need further investigation. Evidence suggests that HMGB1 plays a critical role in the pathogenesis of psoriasis, and HMGB1-based therapeutic strategies may be useful in psoriasis^12–14^. Therefore, the current study aimed to test the hypothesis that LXA4 and its analog can alleviate LPS-induced inflammation in NHEKs and IMQ-induced psoriasiform dermatitis by preventing the expression and translocation of HMGB1.

To confirm the inhibitory effect of LXA4 in psoriasis, IMQ-induced psoriasis in mouse skin was utilized as a model of human psoriasis^23^. We found that pretreatment with BML-111, an agonist of LXA4, attenuated IMQ-induced psoriasiform dermatitis, as evidenced by the improvement in morphological and histopathological features. This suggests that BML-111 is able to protect mice against IMQ-induced skin inflammation, and, therefore, it may be used to treat psoriasis.

Several clinical studies have indicated that inflammatory mediators, such as IL-1β, TNF-α, IFN-γ and IL-6, play critical roles in the initiation and propagation of inflammatory responses in psoriasis^30, 31^. Consistent with previous studies, our results showed that expression of these inflammatory cytokines significantly increased in the skin lesions and sera of IMQ-treated mice compared with those of the normal group, and similar observations were made regarding the expression of IL-17 and IL-23, which are critical for the development of IMQ-induced psoriasiform dermatitis, whereas the administration of BML-111 significantly downregulated the expression of most of these cytokines. These findings suggest that the protective effects of BML-111 on psoriasis can be partly attributed to the inhibition of cytokine production.

Unlike TNF-α and other early proinflammatory cytokines, HMGB1 participates in the process of inflammation as a late mediator^7, 11^. Elevated HMGB1 levels have been detected in the skin tissues and blood samples of patients with psoriasis, indicating that HMGB1 is involved in psoriasis^12, 13^. Similarly, our findings indicated that the expression of HMGB1 was apparently increased in the skin lesions of IMQ-treated mice. More importantly, we observed that BML-111 could diminish the expression and secretion of HMGB1 and protect against IMQ-induced psoriasiform dermatitis. Several studies have shown that some inflammatory mediators, such as LPS, can lead to the secretion of HMGB1 in many cell types^7, 8^. Previous studies have shown that the secretory mechanism of HMGB1 is mediated by the regulation of acetylation of HMGB1^26, 27^. *In vitro* cell culture using NHEKs have shown that LXA4 can also inhibit the translocation, acetylation and expression of HMGB1 upon activation by LPS. Consequently, our study provides convincing evidence that LXA4 and its analog play important roles in suppressing the expression and translocation of HMGB1 in psoriasis.

In the present study, we observed that BML-111 treatment significantly attenuated HMGB1 translocation in IMQ-treated mice, but the effect of secreted HMGB1 in the skin is unclear. Extracellular HMGB1 can interact with RAGE and TLR2/4 to initiate cellular responses. Several studies have demonstrated that TLR4 interacts with extracellular HMGB1 to activate the NF-κB pathway^32^. Other studies have shown that RAGE is the primary binding receptor for HMGB1, which mediates cytokine activity and that the interaction between HMGB1 and RAGE is involved in the process of inflammation^33–35^. In our study, BML-111 modulated the expression of both TLR4 and RAGE in the IMQ-treated mice. Similarly, our findings indicate that LXA4 affected the levels of TLR4 and RAGE following LPS exposure in NHEKs. It is plausible to speculate that both TLR4 and RAGE may be involved in the HMGB1-induced inflammation in psoriasis.

The interaction of extracellular HMGB1 with TLR4/RAGE activates the MAPK-ERK1/2 and NF-κB signaling pathways, which play key roles in inflammatory responses^32, 36^. It has been demonstrated that the MAPK-ERK1/2 signaling is involved in the development of inflammatory skin disease, and inhibitors of this pathway can ameliorate inflammation in various rodent models of human skin diseases^37^. A similar scenario has been described for NF-κB, where the activation of RAGE can induce downstream signaling by promoting NF-κB expression in inflamed skin of mice^38^. Here, we observed that BML-111 and LXA4 treatment significantly attenuated the phosphorylation of ERK1/2 and nuclear NF-κB pathway effectors in the IMQ-induced skin lesions and LPS-induced NHEKs, respectively. This may be because LXA4 and BML-111 suppress the expression of HMGB1, TLR4 and RAGE as well as the secretion of HMGB1. Deficiency of HMGB1, RAGE or TLR4 reduces the activation of the MAPK-ERK1/2 and NF-κB signaling pathways. These data support the notion that the activation of ERK1/2 and NF-κB pathways are mediated by HMGB1-TLR4/RAGE interactions; however, this effect may be ameliorated by treatment with BML-111 and LXA4.

In conclusion, our present study confirms that LXA4 and its analog have protective effects on psoriasiform dermatitis. Administration of BML-111 or LXA4 results in a marked reduction in the translocation and expression of HMGB1. BML-111 downregulates the levels of inflammatory cytokines *in vivo*. These results are consistent with our previous findings, which have shown that LXA4 inhibits the expression of inflammatory cytokines *in vitro* ^17^. Moreover, we have found that LXA4 and BML-111 can suppress the expression of RAGE, TLR4, p-ERK1/2 and NF-κB p65, which are the downstream signaling factors of HMGB1. Our findings suggest that LXA4 and its analog may be potential therapeutic candidates that could be used for psoriasis treatment.

## Methods

### Animals

Female BALB/c mice (18–20 g) were purchased from Beijing Huafukang Biological Technology Co., Ltd. The animals were housed for at least 1 week in a controlled environment (23 ± 2 °C, 12-hour light/dark cycle). Some mice were treated locally with a 42 mg daily dose of IMQ (5%) cream (Mingxin Pharmaceuticals, Sichuan, China) on shaved dorsal skin for 7 consecutive days. On day 8, skin and blood samples were harvested for analysis. All experimental protocols were performed in accordance with the ARRIVE guidelines and were approved by the Ethics Committee for experimental animals, Tongji Medical College (No. 13, Hangkong Road, Wuhan, P.R. China).

### Grouping and treatment

BALB/c mice were randomized into three groups: (1) IMQ + BML-111 group (n = 8), mice received intraperitoneal injection of BML-111 dissolved in saline at a dose of 1 mg/kg/day followed by the application of IMQ cream for 7 days, as described above. (2) IMQ group (n = 8), IMQ-treated mice received injection of the same volume of saline as in the IMQ + BML-111 group for 7 days. (3) Normal group (n = 8), weight-matched mice received intraperitoneal injection of saline and were topically treated with Vaseline (Fig. 1A).

### Scoring of the severity and histology of skin inflammation

To score the severity of the dorsal skin inflammation in mice, an objective scoring system was developed based on the clinical Psoriasis Area and Severity Index (PASI), although the affected skin area was not considered in the overall score. Once daily, the degrees of erythema, scaling and thickening were evaluated on the dorsal skin using a five-point scale: 0, none; 1, slight; 2, moderate; 3, marked; 4, very marked. The PASI score was calculated by adding the scores for the 3 separate criteria, thus allowing for a range from 0 to 12. The scores of each group were then averaged, and trend lines were generated to display the changes in the skin lesions.

On day 8, serum samples and skin tissues were harvested from the mice for subsequent studies. Part of the excised skin was fixed in 10% formalin and embedded in paraffin, and tissue slices of 5-μm thickness were stained with HE for pathological observation under a light microscope. The thickness of the epidermis was measured at four random sites on each tissue strip and quantitated by ImageJ software. Another part of the excised skin was stored at −80 °C for future use.

### Immunofluorescence

The expression of HMGB1 in the lesional skin of mice was detected by immunofluorescence. Immunofluorescence staining was performed as follows: the skin tissues were incubated with primary antibodies against HMGB1 (1:200, Abcam, Cambridge, MA, USA) at 4 °C overnight, followed by incubation with fluorescein isothiocyanate-conjugated secondary antibody for 1 hour. The nuclei were counterstained with 4′,6-diamidino-2-phenylindole (DAPI) for 5 minutes.

### Isolation, culture and stimulation of normal human epidermal keratinocytes

Normal human epidermal keratinocytes (NHEKs) were prepared from healthy individuals who underwent circumcision after informed consent. After removal of subcutaneous tissue and most of the reticular dermis, the tissues were cut into strips and incubated with dispase (grade II, Roche Diagnostics, Mannheim, Germany) at 4 °C overnight. After incubation, the epidermis was separated and enzyme digestion with trypsin (0.25% trypsin with 0.53 mM EDTA; HyClone, Logan, UT, USA) was carried out for 10 minutes. After being neutralized with equal volumes of DMEM containing 10% fetal calf serum (Gibco, Grand Island, NY, USA), the cuticle debris were filtered through nylon mesh to obtain a single cell suspension. The keratinocytes were then cultured in keratinocyte basal medium (Promocell, Heidelberg, Germany) containing a supplement mix of bovine pituitary extract, 0.004 mg/ml; epidermal growth factor (recombinant human), 0.125 ng/ml; insulin (recombinant human), 5 μg/ml; hydrocortisone, 0.33 μg/ml; epinephrine, 0.39 μg/ml; holo-transferrin (human), 10 μg/ml; and CaCl_2_, 0.06 mM. For analysis, the NHEKs were exposed to LPS (10 μg/ml) with and without preincubation with LXA4 (100 nmol/L) for 30 minutes. After 24 hours of incubation, the cell samples and culture supernatant were harvested for further analysis. LXA4 and LPS were purchased from Cayman Chemical (Ann Arbor, MI, USA) and Sigma Chemical company(Saint Louis, MO, USA), respectively. All of the experimental procedures were approved by the ethics review board of Huazhong University of Science and Technology and carried out according to the principles of the Declaration of Helsinki.

### Co-immunoprecipitation

Cultured keratinocytes were lysed with pre-chilled IP buffer, and the supernatant was collected by centrifugation. Part of the cell lysate was denatured and then used as the input. The protein concentration was measured with a BCA protein assay kit. Protein A/G-agarose beads were added to the cell lysate and incubated for 1 hour at 4 °C. The supernatant was collected by centrifugation. Approximately 1.0 µg of antibodies against acetyl-lysine (1:300, Abcam, USA) was added, and the samples were incubated overnight at 4 °C; normal mouse IgG was used as a negative control. Protein A/G-agarose beads were added and incubated for 2 hours. Then, the immunoprecipitated complex was collected by centrifugation and washed 4 times, after that loading buffer was added. The samples were subjected to western blotting analysis.

### Western blot analysis

Total protein samples from tissues or cells were obtained using cell lysis buffer followed by centrifugation at 12000 rpm for 5 minutes at 4 °C. Cytoplasmic and nuclear proteins were isolated using a commercially available Nuclear and Cytoplasmic Protein Extraction Kit (Beyotime Corporation, Shanghai, China) according to the manufacturer’s instructions. The concentrations of proteins in the supernatants were measured using a BCA protein assay kit. Equal amounts of protein were separated by SDS-PAGE gel electrophoresis and then transferred to PVDF membranes. After blocking with 5% BSA for 1 hour at room temperature, the membranes were incubated with primary antibodies against GAPDH (1:1000; Affinity Biosciences, USA), Lamin B (1:200; BOSTER Corporation, Wuhan, China), HMGB1 (1:10000; Abcam, USA), RAGE (1:200; Santa Cruz, USA), TLR4 (1:200; Santa Cruz, USA), NF-κB p65 (1:1000; Cell Signaling Technology, USA), p-ERK1/2 (1:2000; Cell Signaling technology, USA), and ERK1/2 (1:1000; Cell Signaling technology, USA) at 4 °C overnight and subsequently with the appropriate HRP-conjugated secondary antibodies for 1 hour at room temperature. The immunoreactive bands were visualized using a DNR bio-imaging system according to the manufacturer’s instructions. The expression of cytoplasmic protein was normalized to GAPDH, and the expression of nuclear proteins was normalized to Lamin B using ImageJ software.

### Real-time quantitative PCR

Total mRNA was extracted from keratinocytes and cells of the dorsal skin samples with TRIzol Reagent (Takara Biotechnology, Otsu, Japan). After extraction, reverse transcription from total RNA to cDNA was performed with a PrimeScript RT Reagent Kit (Takara Biotechnology, Otsu, Japan) according to the manufacturer’s protocols. PCR was performed with an RNA PCR kit (Takara Biotechnology, Otsu, Japan). The 2^−ΔΔCT^ method was used to quantitatively analyze the data. The PCR primer sequences are given in Supplementary Table S1.

### Enzyme-linked immunosorbent assay (ELISA)

The concentration of IL-1β, TNF-α, IL-23 and IL-17 in the sera of BALB/c mice and that of HMGB1 in the cell culture media were detected by ELISA according to the manufacturer’s instructions (ELISA kits for IL-1β and IL-17: eBioscience, Frankfurt, Germany; ELISA kits for TNF-α and IL-23: R&D systems, Minneapolis, MN, USA; ELISA kit for HMGB1: IBL, Hamburg, Germany). All assays were performed in duplicate.

### Statistical analysis

The data were expressed as the mean ± SD, and GraphPad Prism v. 6.0 software was used for statistical analyses. Student’s *t*-test or one-way analysis of variance (ANOVA) was used to determine the statistical significance of differences. A value of *P* < 0.05 was considered statistically significant.

## Electronic supplementary material

Supplementary Information
