# Supplementary material for: Lipoxin A4 and its analog suppress inflammation by modulating HMGB1 translocation and expression in psoriasis
Source: Sci Rep. 2017 Aug 2;7:7100. doi: 10.1038/s41598-017-07485-1 (PMC5541073; doi:10.1038/s41598-017-07485-1)
Supplement: Supplementary file 1 — Supplementary Information [file 41598_2017_7485_MOESM1_ESM.pdf]

Supplementary Information

Lipoxin A4 and its analog suppress inflammation by modulating HMGB1 translocation and expression in psoriasis

Author list and affiliations:

Xinxin Liu[1*], Xin Wang[1*], Xiaoru Duan[1*], Devesh Poorun[1], Juntao Xu[1], Song Zhang[1], Lu Gan[1], Mengwen He[1], Ke Zhu[1], Zhangyin Ming[3], Feng Hu[1], Hongxiang Chen[1,2]

1 Union Hospital, Tongji Medical College, Huazhong University of Science and Technology, Wuhan 430022, China

2 Cutaneous Biology Research Center, Department of Dermatology, Massachusetts General Hospital, Harvard Medical School, Building 149, 13th Street Charlestown, Boston, MA,02129,USA

3 Department of Pharmacology, Tongji Medical College, Huazhong University of Science and Technology, Wuhan 430030, China

*These authors contributed equally to this work.

Correspondence and requests for materials should be addressed to H.C.

(email: hongxiangchen@hotmail.com)

Supplementary Figure S1. Full-size blots of Fig. 2B

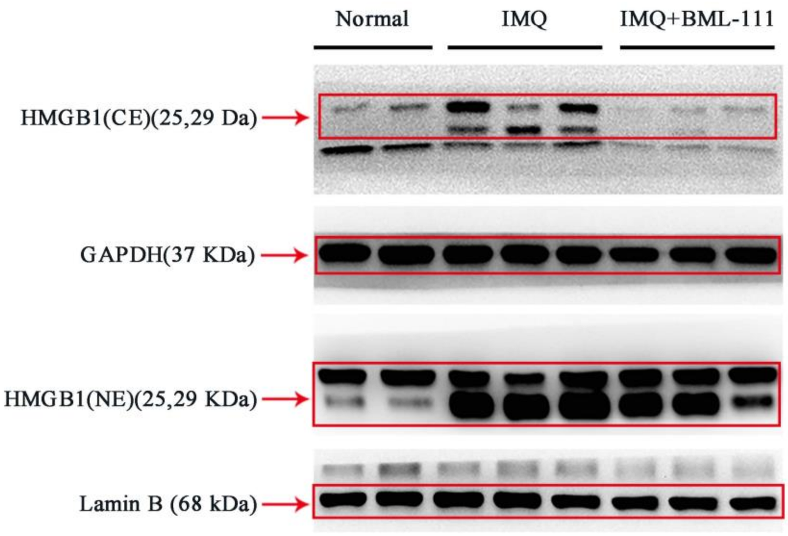

Supplementary Figure S2. Full-size blots of Fig. 3A

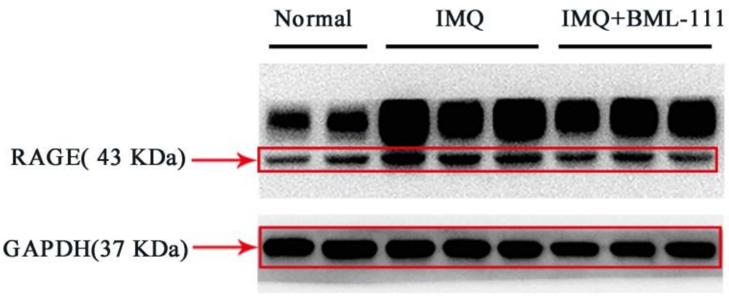

Supplementary Figure S3. Full-size blots of Fig. 3D

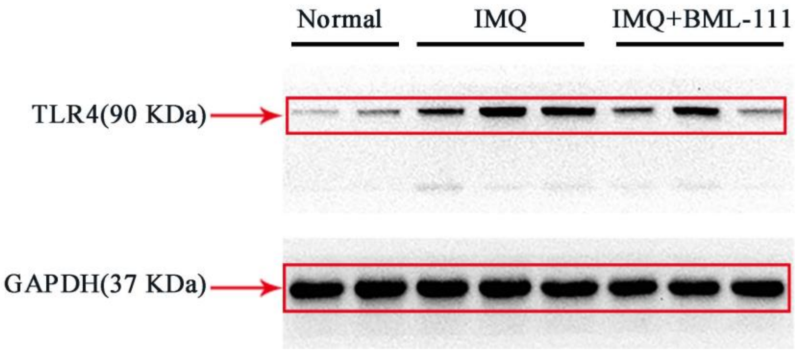

Supplementary Figure S4. Full-size blots of Fig. 3G

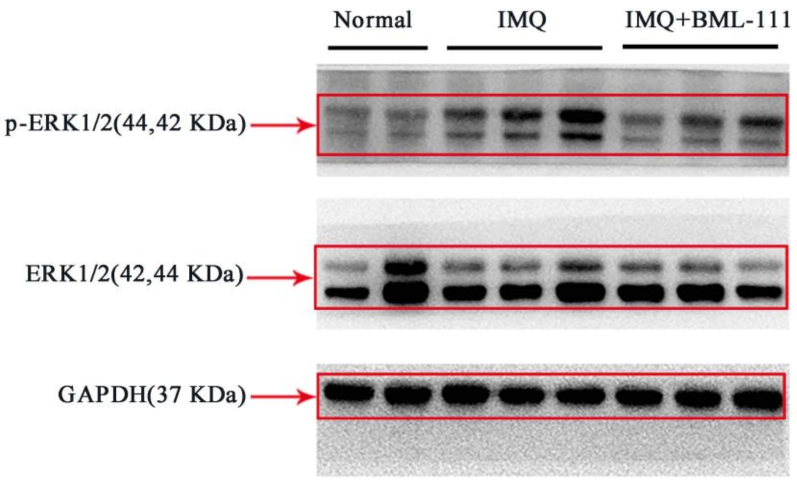

Supplementary Figure S5. Full-size blots of Fig. 3I

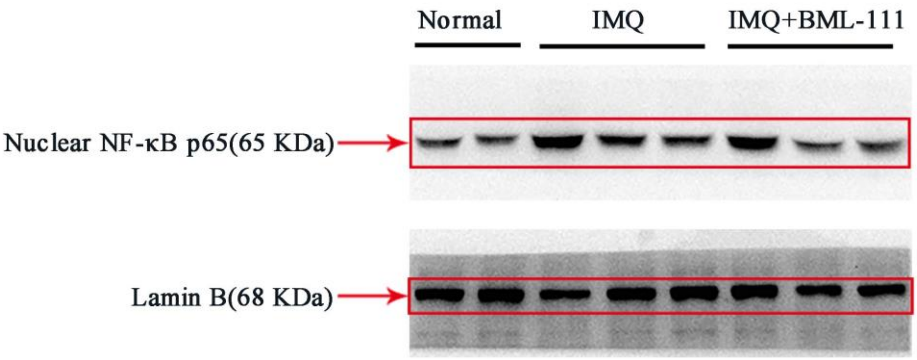

Supplementary Figure S6. Full-size blots of Fig. 5A

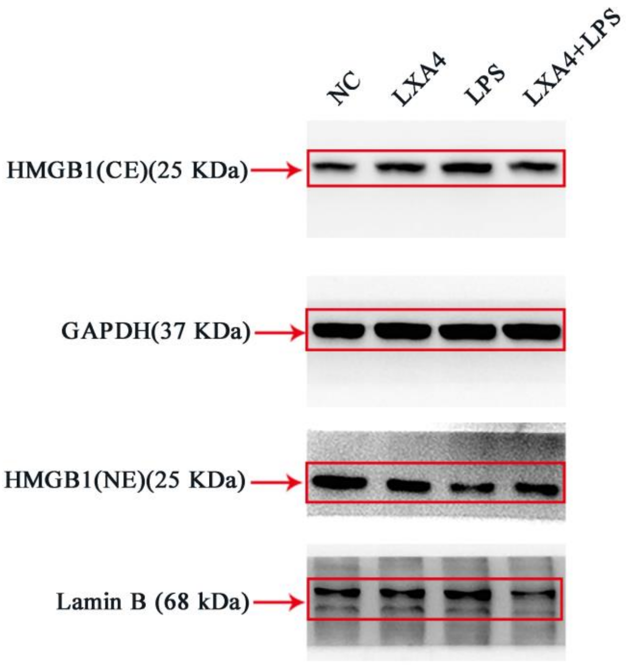

Supplementary Figure S7. Full-size blots of Fig. 5D

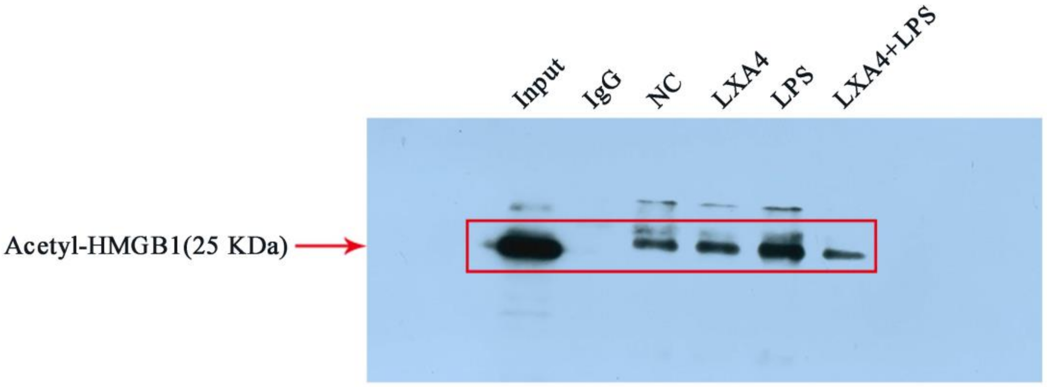

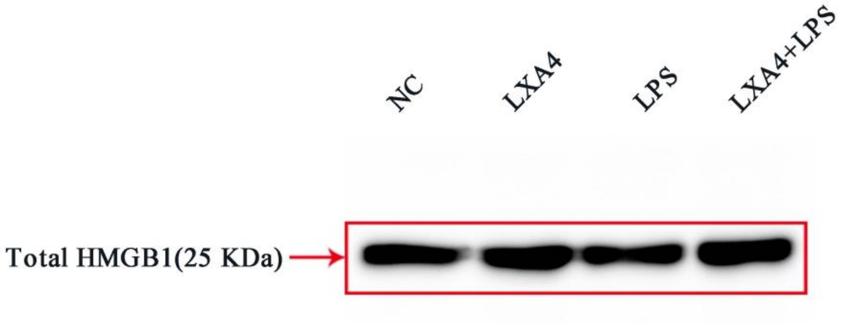

Supplementary Figure S8. Full-size blots of Fig. 6A

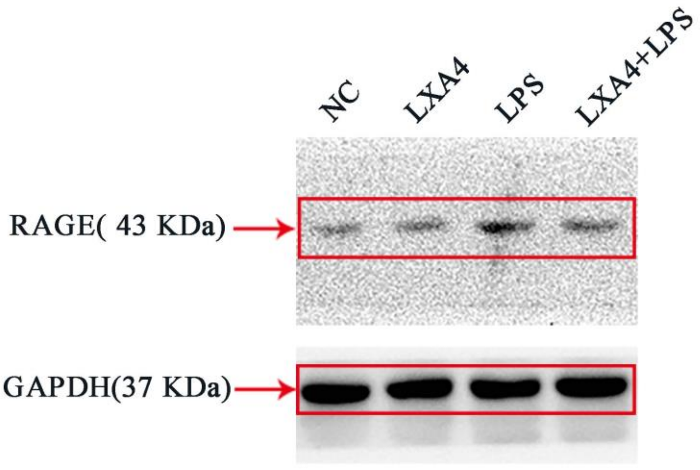

Supplementary Figure S9. Full-size blots of Fig. 6D

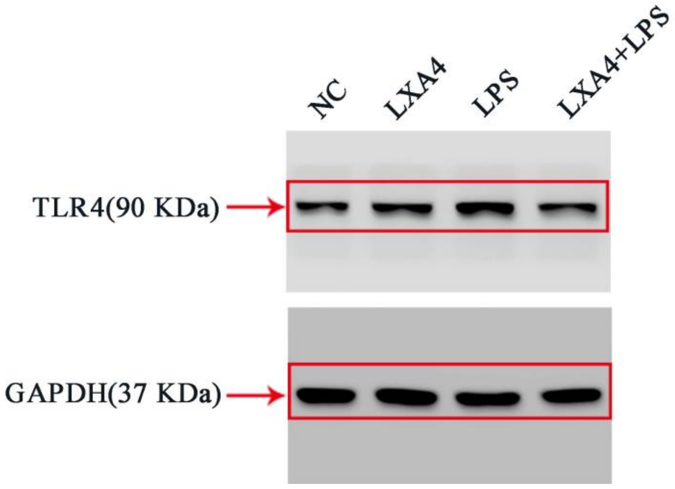

Supplementary Figure S10. Full-size blots of Fig. 6G

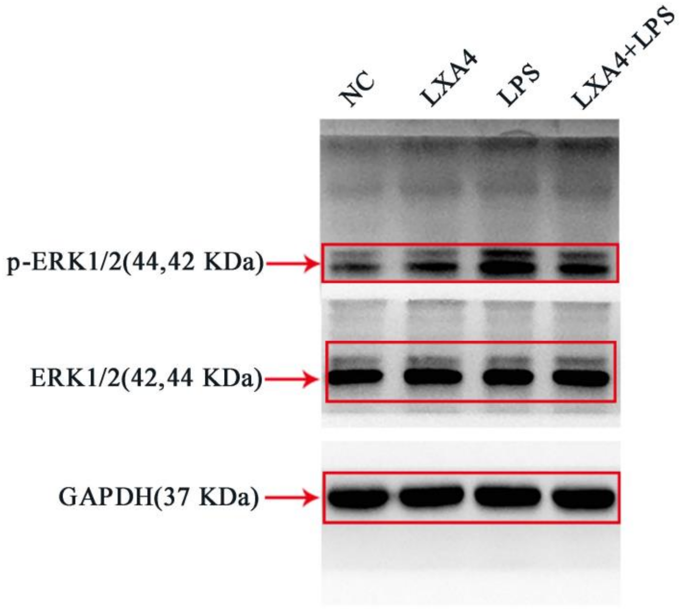

Supplementary Figure S11. Full-size blots of Fig. 6I

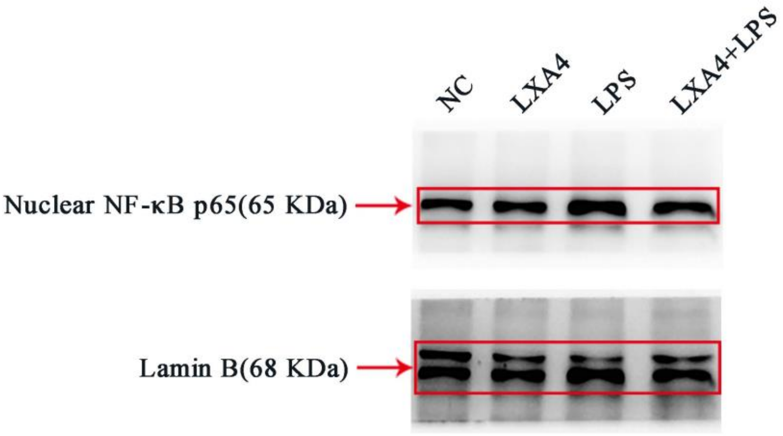

Supplementary Table S1 : Primers for real-time quantitative PCR.

Gene name		5'- 3' primer sequence
MusGAPDH	Forward	TCTCCTGCGACTTCAACA
	Reverse	TGTAGCCGTATTCATTGTCA
MusHMGB1	Forward	AGGAAGATGATGAGGAGGAT
	Reverse	GGCAAGGTTAGTGGCTATT
MusRAGE	Forward	GGAAGAGACCAGGAGACA
	Reverse	CACCAGGAGCGACTATTC
MusTLR4	Forward	TTCACCTCTGCCTTCACT
	Reverse	GGACTTCTCAACCTTCTCAA
MusIL-1β	Forward	CTTCAGGCAGGCAGTATC
	Reverse	CAGCAGGTTATCATCATCATC
MusIL-6	Forward	CCGCTATGAAGTTCCTCTC
	Reverse	GGTATCCTCTGTGAAGTCTC
MusTNF-α	Forward	TGTCCATTCCTGAGTTCTG
	Reverse	GGAGGCAACAAGGTAGAG
Mus IFN-γ	Forward	ATGAACGCTACACACTGCATC
	Reverse	CCATCCTTTTGCCAGTTCCTC
MusIL-17a	Forward	ACTACCTCAACCGTTCCA
	Reverse	GAATCTGCCTCTGAATCCA
MusIL-17c	Forward	ATGCTTGTGTCGTGGATG
	Reverse	GTGCCTGGAATGTCTGTC
MusIL-23	Forward	ACCTGCTTGACTCTGACA
	Reverse	CCACTGCTGACTAGAACTC
MusIL-22	Forward	ATGAGTTTTTCCCTTATGGGGAC
	Reverse	GCTGGAAGTTGGACACCTCAA

HomoGAPDH	Forward	GGCTCTCCAGAACATCATC
	Reverse	TCTTCCTCTTGTGCTCTTG
HomoHMGB1	Forward	TCTTCCTCTTCTGCTCTGA
	Reverse	ATCTTCCTCCTCTTCCTTCT
HomoRAGE	Forward	CCTGGTGCCTAATGAGAAG
	Reverse	GATGATGCTGATGCTGACA
HomoTLR4	Forward	TCCTTCACTACAGAGACT
	Reverse	CTTCAGATAGATGTTGCTTCC
